# The BRAIN TRIAL: a randomised, placebo controlled trial of a Bradykinin B2 receptor antagonist (Anatibant) in patients with traumatic brain injury

**DOI:** 10.1186/1745-6215-10-109

**Published:** 2009-12-03

**Authors:** Haleema Shakur, Peter Andrews, Toomas Asser, Laura Balica, Cristian Boeriu, Juan Diego Ciro Quintero, Yashbir Dewan, Patrick Druwé, Olivia Fletcher, Chris Frost, Bennie Hartzenberg, Jorge Mejia Mantilla, Francisco Murillo-Cabezas, Jan Pachl, Ramalingam R Ravi, Indrek Rätsep, Cristina Sampaio, Manmohan Singh, Petr Svoboda, Ian Roberts

**Affiliations:** 1 Department of Epidemiology and Population Health, London School of Hygiene & Tropical Medicine, Keppel Street, London WC1E 7HT, UK; 2 Department of Anaesthesia, Critical Care & Pain Medicine, University of Edinburgh, Royal Infirmary, Little France, Edinburgh EH16 4SA, UK; 3 Department of Neurology and Neurosurgery, University of Tartu, 2 Ludvig Puusepp St, 51014 Tartu, Estonia; 4 Emergency Department, Spitalul Clinic de Urgenţă Bucureşti, 8 Floreasca Street, Sector 1, 014461 Bucharest, Romania; 5 Mobile Emergency Service, Spitalul Clinic Judetean Mureş, 50 Gheorghe Marinescu Street, 540103 Târgu Mureş, Romania; 6 Department of Critical Care, Clínica las Americas, Diagonal 75B # 2A-80 UCI, Medellin, Colombia; 7 Neurology Clinic, Instituto Neurológico de Antioquia, Calle 55 # 46-36 UCI, Medellin, Colombia; 8 Department of Neurosurgery, Christian Medical College, Ludhiana, Punjab 141008, India; 9 Department of Accident and Emergency/Intensive Care, Sint-Vincentiushospital, Sint-Vincentiusstraat 20, Antwerp 2018, Belgium; 10 Department of Neurosurgery, Tygerberg Academic Hospital, Francie van Zijl Drive, Tygerberg 7505, South Africa; 11 Department of Intensive Care and Anaesthesia, Fundación Clínica Valle del Lili, Cra 98 No 18-49, Cali, Valle del Cauca, Colombia; 12 Department of Emergency and Intensive Care, Hospital Universitario Virgen del Rocio, Avda Manuel Siurot S/N, 41013 Seville, Spain; 13 Department of Anaesthesiology and CCM, Hospital Královské Vinohrady, Šrobárova 50, Prague 10, 100 34, Czech Republic; 14 Medical Trust Hospital, M G Road, Ernakulam, Kochi 682016, Kerala, India; 15 Department of Postoperative Intensive Care, North Estonian Regional Hospital, Sütiste tee 19, 13419 Tallinn, Estonia; 16 Laboratório de Farmacología Clínica e Tereapêutica, Faculdade de Medicina de Lisboa, Edifício do Hospital de Santa Maria, 1629-049 Lisbon, Portugal; 17 Department of Neurosurgery, All India Institute of Medical Sciences, Neurosciences Centre, Ansari Nagar, New Delhi 110029, India; 18 Research Institute for Special Surgery and Trauma, Ponavka 6, 662 50 Brno, Moravia, Czech Republic

## Abstract

**Background:**

Cerebral oedema is associated with significant neurological damage in patients with traumatic brain injury. Bradykinin is an inflammatory mediator that may contribute to cerebral oedema by increasing the permeability of the blood-brain barrier. We evaluated the safety and effectiveness of the non-peptide bradykinin B2 receptor antagonist Anatibant in the treatment of patients with traumatic brain injury. During the course of the trial, funding was withdrawn by the sponsor.

**Methods:**

Adults with traumatic brain injury and a Glasgow Coma Scale score of 12 or less, who had a CT scan showing an intracranial abnormality consistent with trauma, and were within eight hours of their injury were randomly allocated to low, medium or high dose Anatibant or to placebo. Outcomes were Serious Adverse Events (SAE), mortality 15 days following injury and in-hospital morbidity assessed by the Glasgow Coma Scale (GCS), the Disability Rating Scale (DRS) and a modified version of the Oxford Handicap Scale (HIREOS).

**Results:**

228 patients out of a planned sample size of 400 patients were randomised. The risk of experiencing one or more SAEs was 26.4% (43/163) in the combined Anatibant treated group, compared to 19.3% (11/57) in the placebo group (relative risk = 1.37; 95% CI 0·76 to 2·46). All cause mortality in the Anatibant treated group was 19% and in the placebo group 15.8% (relative risk 1.20, 95% CI 0.61 to 2.36). The mean GCS at discharge was 12.48 in the Anatibant treated group and 13.0 in the placebo group. Mean DRS was 11.18 Anatibant versus 9.73 placebo, and mean HIREOS was 3.94 Anatibant versus 3.54 placebo. The differences between the mean levels for GCS, DRS and HIREOS in the Anatibant and placebo groups, when adjusted for baseline GCS, showed a non-significant trend for worse outcomes in all three measures.

**Conclusion:**

This trial did not reach the planned sample size of 400 patients and consequently, the study power to detect an increase in the risk of serious adverse events was reduced. This trial provides no reliable evidence of benefit or harm and a larger trial would be needed to establish safety and effectiveness.

**Trial Registration:**

This study is registered as an International Standard Randomised Controlled Trial, number ISRCTN23625128.

## Background

Cerebral oedema is associated with significant mortality and morbidity after traumatic brain injury (TBI). It develops soon after injury, reaching a maximum between 3 and 5 days post injury [1]. An increase in the permeability of the blood-brain barrier (BBB) is believed to be responsible for oedema formation. Bradykinin (BK), an inflammatory mediator in the kinin-kallikrein system, may contribute to cerebral oedema by increasing permeability of the BBB. BK is produced immediately after injury and appears to be a potent endogenous disruptor of the BBB [2]. BK increases the permeability of the BBB to small solutes and increases blood pressure in the microcirculation due to arterial vasodilatation and venous constriction [3]. Levels of BK1-5, a stable circulating metabolite of systemic BK in humans, have been shown to decrease steeply within the first 80 hours after TBI [4].

Bradykinin B2 receptor antagonists (BB2) block the activity of the kinin-kallikrein system, preventing the release of BK, and may reduce neuronal damage after TBI [5]. A systematic review of controlled studies in animal brain injury models showed that BB2 antagonists reduce brain oedema and improve neurological outcome [6]. A systematic review of randomised trials of BB2 antagonists in TBI patients found three small trials with a total of 178 participants and provided no reliable evidence of either benefit or harm. There were, however, non-significant reductions in mortality and disability with BB2 antagonists [7].

Anatibant, a non-peptide, selective BB2 antagonist, reduces brain oedema in animal TBI models [8-11]. No serious toxicity was found in Phase 1 clinical trials [4,12]. Following subcutaneous administration of clinically relevant doses, there were no systemic effects but there was pain, inflammation and nodule formation at the injection site.

Anatibant inhibits the binding of BK to the B2 receptor. After subcutaneous injection Anatibant is bio-available and crosses the BBB. Pharmacokinetic (PK) studies show brain interstitial fluid levels in excess of the concentration required to produce half maximum inhibition at the B2 receptor. Anatibant has a plasma half-life after subcutaneous administration of 30 and 70 hours in healthy volunteers and TBI patients respectively and can be given as a once daily subcutaneous injection. Metabolism and excretion is mainly through hepatic and biliary routes. PK parameters are increased 1.5-2 fold in TBI patients compared to healthy volunteers, possibly due to decreased hepatic clearance [4,12].

Because plausible treatment effects in TBI are likely to be modest, we planned to conduct a large phase III trial with at least five thousand patients. As a prelude to this, we set out to conduct a phase II trial of 400 patients to assess safety and to inform the dose selection for the phase III trial.

## Methods

All collaborating investigators were required to secure the relevant ethics and regulatory approvals before recruitment could begin. Consent was obtained in accordance with the requirements of each ethics committee.

### Eligibility

All non-pregnant adults (ages 16 to 65 years inclusive) with a traumatic brain injury, who had a score on the Glasgow Coma Scale (GCS) of 12 or less (out of a maximum score of 15) and who had a CT scan showing an intracranial abnormality consistent with trauma, and were within eight hours of their injury, were eligible for trial entry if the responsible doctor considered there was the potential for them to benefit from the trial treatment. Patients known to have been treated with another investigational drug therapy within 30 days of injury were excluded.

### Intervention

Patients were randomly allocated to receive a low (10 mg loading dose and 5 mg/day), medium (20 mg loading dose and 10 mg/day) or high (30 mg loading dose and 15 mg/day) dose of Anatibant or matching placebo. The loading dose was given as soon as possible and within 8 hours of injury and administered as two simultaneous subcutaneous injections of 5 mg, 10 mg and 15 mg respectively. The maintenance dose was given 24 hours after the loading dose and continued daily for 4 days.

### Study objectives

The primary study objective was to compare the proportion of patients with at least one SAE in those receiving Anatibant and those receiving placebo. The secondary study objectives were to assess the effect of Anatibant on early mortality and morbidity and to establish the dose to be used in the phase III trial. Samples for pharmacokinetic analyses were collected but these data are not reported here.

### Study outcomes

A Serious Adverse Event (SAE) was defined as any untoward medical occurrence that was (1) fatal; (2) life threatening; (3) required or prolonged hospitalisation; (4) resulted in persistent or significant disability or incapacity; (5) medically significant in that it may jeopardise the patient and may require medical or surgical intervention to prevent one of the outcomes listed above; or (6) congenital anomalies. Medical occurrences that were expected in the course of traumatic brain injury, or which were known to occur during routine diagnostic or therapeutic procedures, were excluded. Any SAE occurring after the administration of the first dose of study medication and up to 15 days following injury was included. Mortality was assessed up to 15 days following the injury. In-hospital morbidity was assessed 15 days after injury or at withdrawal using the Glasgow Coma Scale (GCS), the Disability Rating Scale (DRS) and a modified version of the Oxford Handicap Scale which is referred to here as the Head Injury Related Early Outcome Score (HIREOS).

### Sample size

We planned to randomise a total of 400 patients, 100 to each of the four groups. Assuming that the proportion of patients with at least one SAE would be 20% in the placebo group, the trial would have had 90% statistical power to detect a doubling of this proportion (to 40%) in the combined Anatibant groups using a 1-sided significance level of 5%. A 1-sided test was considered appropriate since a decision to not continue to a Phase III trial would only have been taken if the proportion of patients with at least one SAE had been higher in the combined Anatibant groups.

### Randomisation

Randomisation was done centrally by Sealed Envelope Ltd, UK, using an Interactive Voice Response System (IVRS). Initial patient information confirming eligibility was first collected via the IVRS. The central study computer then randomly assigned a treatment pack number corresponding to one of a number of blinded treatment packs available in the emergency department of the participating hospital. The information collected was used to achieve balance with respect to key prognostic factors using minimisation on the basis of the following variables: sex, age (16 to ≤40; >40 to ≤50; >50 to ≤65), time since injury: (≤1 h, >1 to ≤3 h, >3 to ≤8 h); GCS: 3 to 5; 6 to 8; 9 to 12; and pupil reactivity (both, one or none).

### Blinding

Active drug and placebo were visually matched and packed by an independent clinical trials supply company, Bilcare, UK. All study personnel and participants were to be blinded to treatment assignment for the duration of the study. Only the independent Data Safety Monitoring Board (DSMB) and their independent statistician were to see un-blinded data but none had any contact with study participants.

### Statistical methods

All data analyses were carried out according to a pre-defined statistical analysis plan. Both intention-to-treat and per-protocol analyses were undertaken. As per the analysis plan the primary analyses reported here compare results in the combined Anatibant groups with those in the placebo group. Because the trial stopped early, formal statistical comparisons between dose groups, although specified in the analysis plan, are not reported here. In addition, for the updated meta-analysis of effect of Bradykinin B2 receptor antagonists on mortality after TBI, the relative risks (RR) and 95% confidence intervals (CIs) for mortality were calculated for each trial and pooled using the fixed-effect model.

The proportion of patients with at least one SAE in the combined Anatibant groups was compared with that in the placebo group using a Fisher's exact test. The relative risk and 95% confidence interval were also calculated. The result was declared significant if the p-value was less than 0.05 (1-sided test).

All cause mortality in the period up to and including 15 days post randomisation was compared using Fisher's exact test. GCS, DRS and HIREOS scores at 15 days post randomisation were compared using an analysis of covariance model to adjust for baseline GCS scores. Since these variables are not normally distributed, non-parametric bias-corrected and accelerated 95% bootstrap confidence intervals (2,000 replications) were computed and used to infer statistical significance at the 5% level [13]. No subgroup analyses were planned or performed because it was considered that there would be insufficient power to detect plausible levels of interaction.

### Safety Monitoring

During the trial, interim analyses of all data including SAEs, mortality and morbidity were carried out by an independent DSMB. The DSMB had the responsibility for deciding whether, while recruitment was in progress, the un-blinded results (or the un-blinded results for a particular subgroup), should be revealed to the Trial Steering Committee (TSC). Because the trial was collecting data on a range of potential safety end points and it would have been impossible to specify in advance the potential adverse effects, there were no formal stopping rules. Instead, the DSMB was required to reveal the un-blinded results to the TSC if, taking into account both statistical and clinical issues and exercising their best clinical and statistical judgement, the un-blinded results provided sufficient evidence that the trial treatment was on balance harmful for all, or for a particular category of patients.

### Role of the funding source and other relevant organisations

Xytis Pharmaceutical Sarl (Switzerland) was legal sponsor and funder and was involved in the development of the protocol. Xytis had one non-voting representative on the TSC but did not contribute to this data analysis. The TSC provided overall supervision of the trial. In particular, the TSC concentrated on the progress of the trial, adherence to the protocol, patient safety and consideration of new information. The trial was coordinated and managed by the London School of Hygiene & Tropical Medicine (LSHTM).

## Results

The first patient was enrolled in March 2007. On 1 November 2007, after reviewing un-blinded data on 140 patients, the DSMB disclosed limited un-blinded results to the TSC because of patient safety concerns. By that time a total of 228 patients had been randomised. The TSC suspended recruitment until a review of the data on all 228 patients could be undertaken by the DSMB. Presentation of this data to the DSMB was delayed due to a legal dispute between LSHTM and Xytis which was heard in the High Court of London in March 2008. The DSMB finally reviewed the data on 6 June 2008 and recommended that recruitment could recommence. After funding was withdrawn the trial was terminated.

In the course of the trial a number of protocol deviations occurred, including LSHTM not receiving all trial data and information on SAEs. LSHTM withdrew Clinical Research Associates' (CRA) access to the clinical trial database for a period of time when this occurred. In addition, the High Court of Justice in London granted an injunction preventing the TSC or the independent DSMB from making any determination or recommendation to terminate the trial. The Sponsor obtained the randomisation code from the company responsible for drug packing and conducted three un-blinded analyses on different data sets prior to the termination of the trial. In March 2008, by order of the High Court of Justice, all claims against the LSHTM were dismissed and the injunctions were discharged. All public domain documentation is available on the trial coordinating centre web-site http://www.trialscoordinatingcentre.Lshtm.ac.uk/.

### Randomisations

**Figure 1:**
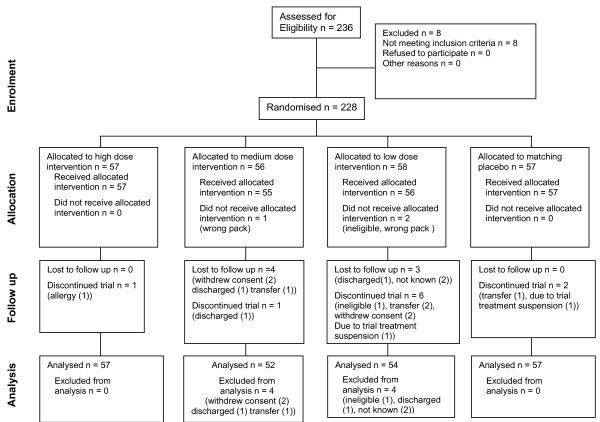
**CONSORT Flowchart**. Patients who withdrew from the trial and for whom final outcome measurements (GCS, HIREOS and DRS) were obtained, but earlier than scheduled, are classified as having ***discontinued the trial***. Patients who withdrew from the trial without completing their final outcome measurements are classified as ***lost to follow up***. Individuals who were ***lost to follow up ***do not contribute to the analyses; those who ***discontinued the trial ***contribute to this intention to treat analysis if data were available.

The 228 patients randomised to Anatibant or placebo group (Figure 1) up to 1 November 2007 were recruited from 15 hospitals in eight countries. There were protocol violations in 11 patients. One patient had a normal CT scan and therefore did not meet the inclusion criteria; two patients were later found to be legal minors; three patients received the trial treatment more than 8 hours after injury; two patients did not receive the allocated treatment; one patient did not complete the locally required consent procedure; one patient was given the trial treatment in the wrong injection site (shoulder rather than abdomen or thigh) and in one patient administration of the maintenance dose was delayed. A total of 219 patients were randomised using the IVRS method and 9 used a back-up system which involved selecting the lowest numbered treatment pack available at the hospital. The back-up system was used to randomise patients for whom the trial coordinating centre had granted a protocol pre-specified waiver to one of the eligibility criteria, and in the event of failure of the international free-phone system. Treatment groups were approximately balanced with respect to baseline characteristics (Table 1).

**Table 1:** Baseline Characteristics.

	High dose XY2405	Medium dose XY2405	Low dose XY2405	All doses XY2405 combined	Placebo
Patients randomised: n	57	56	58	171	57

Age:					
mean (SD)	35.9 (14.0)	37.6 (14.3)	35.5 (13.8)	36.3 (14.0)	36.4 (14.3)
median (min -- max)	33 (19 -- 65)	37 (18 -- 64)	32 (17 -- 61)	34 (17 -- 65)	34 (17 -- 64)
16 -- 39 (%)	36 (63.2)	32 (57.1)	35 (60.3)	103 (60.2)	35 (61.4)
40 -- 49 (%)	8 (14.0)	11 (19.6)	9 (15.5)	28 (16.4)	8 (14.0)
50 + (%)	13 (22.8)	13 (23.2)	14 (24.1)	40 (23.4)	14 (24.6)

Gender: n (%)					
male	51 (89.5)	50 (89.3)	52 (89.7)	153 (89.5)	50 (87.7)

Time since injury:					
mean (SD)	5.5 (1.7)	5.7 (1.9)	5.8 (1.8)***	5.7 (1.8)	5.9 (1.6)
median (min -- max)	5.8 (1.4 -- 8.0)	5.8 (2.1 -- 10.8)*	5.8 (1.8 -- 8.6)*	5.8 (1.4 -- 10.8)*	6.2 (2.3 -- 8.0)
≤ 1 hr (%)	0	0	0	0	0
> 1hr - ≤ 3 hrs (%)	4 (7.0)	3 (5.4)	5 (8.6)	12 (7.0)	4 (7.0)
> 3hrs - ≤ 8 hrs (%)	53 (93.0)	52 (92.9)	50 (86.2)	155 (90.6)	53 (93.0)
> 8 hrs (%)	0	1 (1.8)	2 (3.4)	3 (1.8)	0

Glasgow Coma Score:					
mean (SD)	7.4 (3.0)	7.7 (3.4)	7.6 (2.9)	7.6 (3.1)	7.5 (3.0)
median (min-max)	7 (3 -- 14)**	7 (3 -- 15)**	7 (3 -- 12)	7 (3 -- 15)**	8 (3 -- 12)
3 -- 5 (%)	17 (29.8)	15 (26.8)	17 (29.3)	49 (28.7)	18 (31.6)
6 -- 8 (%)	18 (31.6)	19 (33.9)	17 (29.3)	54 (31.6)	17 (29.8)
9 -- 12 (%)	21 (36.8)	21 (37.5)	24 (41.4)	66 (38.6)	22 (38.6)
>12 (%)	1 (1.8)	1 (1.8)	0	2 (1.2)	0

Glasgow Coma Motor Sub-score: n					
mean (SD)	3.8 (1.5)	3.9 (1.8)	4.1 (1.5)***	3.9 (1.6)	4.0 (1.7)
median (min-max)	4 (1 -- 6)	5 (1 -- 6)	5 (1 -- 6)	5 (1 -- 6)	5 (1 -- 6)

Pupil reactivity to light: n (%)					
Not testable (%)	0	0	0	0	0
Neither (%)	11 (19.3)	6 (10.7)	4 (6.9)	21 (12.3)	6 (10.5)
Only one reactive (%)	3 (5.3)	8 (14.3)	11 (19.0)	22 (12.9)	7 (12.3)
Both reactive (%)	43 (75.4)	42 (75.0)	43 (74.1)	128 (74.9)	44 (77.2)

Body Mass Index:					
mean (SD)	24.5 (4.0)	24.0 (2.8)	24.0 (3.1)***	24.2 (3.3)	24.9 (2.9)
median (min-max)	24.2 (17.3 -- 39.4)	24.2 (18.1 -- 31.1)	23.4 (17.3-33.2)	23.9 (17.3 -- 39.4)	24.4 (19.7-34.6)

Temperature:					
mean (SD)	36.7 (0.8)#	36.6 (0.7)#	36.6 (0.8)#	36.6 (0.8)#	36.6 (0.9)#
median (min-max)	36.9 (34.0 -- 38.9)	36.7 (33.7 -- 37.8)	36.8 (33.7 -- 38.3)	36.8 (33.7 -- 38.9)	36.8 (33.5 -- 39.4)

Systolic Blood Pressure:					
mean (SD)	132.6 (26.7)	128.0 (21.4)	130.4 (20.6)***	130.3 (23.0)	124.7 (22.7)
median (min-max)	130.0 (98.0-212.0)	130.0 (91.0-200.0)	129.0 (93.0-190.0)	130.0 (91.0-212.0)	125.0 (60.0-177.0)

* Three individuals had their first injection after 8 hours

** Two individuals had Glasgow coma scores >12 but fulfilled all other eligibility criteria. As per protocol (6.5.2) a waiver was granted to allow these individuals to be randomised.

*** 1 missing value for low dose

# 3 missing values for high dose, 2 missing values for medium dose, 1 missing value for low dose, 1 missing value for placebo

Both per protocol (PP) and intention to treat analyses (ITT) were conducted. PP analysis is presented only for the primary analysis (number and percentage of patients with at least one SAE). ITT analysis is presented for all other analyses since there were no material differences between them.

### Effects on Serious Adverse Events up to Day 15

There were 43 (26.4%) patients with at least one SAE within two weeks of randomisation among the 163 patients allocated Anatibant (all doses combined), compared with 11 (19.3%) among the 57 allocated placebo (Table 2). There was no significant effect of Anatibant on the risk of at least one SAE within two weeks (RR = 1.37 (0.76 to 2.46), 1 sided *p*= 0.19). There were no SAEs suspected to be related to the study drug as judged by investigators.

**Table 2:** Adverse Events and outcomes by intention to treat other than where otherwise indicated.

	High dose XY2405	Medium dose XY2405	Low dose XY2405	All doses XY2405 combined	Placebo
Number of patients	57	56	58	171	57

Number (%) of patients with at least one serious adverse event	15/57 (26.3%)	15/52^# ^(28.8%)	13/54^# ^(24.1%)	43/163^# ^(26.4%) RR = 1.37 (0.76, 2.46) 1 sided P = 0.19	11/57 (19.3%)

Number (%) of patients with at least one serious adverse event (Per Protocol analysis)	15/56 (26.8%)	15/48 (31.3%)	12/46 (26.1%)	42/150 (28.0%) RR = 1.48 (0.80, 2.74) 1 sided P = 0.13	10/53 (18.9%)

Number (%) of patients with at least one serious adverse event which is suspected to be related to study drug	0	0	0	0	0

Number of serious adverse events#					
0	42 (73.3%)	37 (71.2%)	41 (75.9%)	120 (73.6%)	46 (80.7%)
1	12 (21.1%)	8 (15.4%)	11 (20.4%)	31 (19.0%)	7 (12.3%)
2	2 (3.5%)	5 (9.6%)	1 (1.9%)	8 (4.9%)	3 (5.3%)
3+	1 (1.8%)	2 (3.8%)	1 (1.9%)	4 (2.5%)	1 (1.8%)
					
Total number SAEs	19	25	16	60	16

Glasgow Coma Score at day 15	12.54	12.63	12.29	12.48	13.00
	δ = -0.36 P > 0.05 (-1.56, 0.92)	δ = -0.48 P > 0.05 (-1.55, 0.78)	δ = -0.80 P > 0.05 (-2.00, 0.53)	δ = -0.55, P > 0.05 (-1.42, 0.59)	Ref
	10.70*	10.60*	10.74*	10.68* δ = -0.78 P > 0.05 (-2.08, 0.61)	11.42* Ref

HIREOS at day 15	3.98	3.89**	3.92	3.93	3.54
	δ = 0.43 P > 0.05 (-0.17, 1.05)	δ = 0.42 P > 0.05 (-0.19, 0.98)	δ = 0.39 P > 0.05 (-0.21, 0.98)	δ = 0.41 P > 0.05 (-0.06, 0.89)	Ref

Disability Rating Scale at day 15	11.26	10.61**	11.63	11.18	9.73
	δ = 1.17 P > 0.05 (-2.70, 4.60)	δ = 1.46 P > 0.05 (-1.60, 4.80)	δ = 2.22 P > 0.05 (-1.25, 5.66)	δ = 1.62 P > 0.05 (-1.16, 4.23)	Ref
	14.88*	14.63*	14.69*	14.74* δ = 1.97 P > 0.05 (-1.27, 5.00)	12.93* Ref

All cause mortality up to day 15: (%)	11/57 (19.3%)	11/52#(21.2%)	9/54# (16.7%)	31/163# (19.0%) RR = 1.20 (0.61, 2.36) 1 sided P = 0.37	9/57 (15.8%)

Number (%) of patients with erythema at injection site classified as moderate, intense or very intense.	12/57 (21.1%)	3/52# (5.8%)	4/54# (7.4%)	19/163# (11.7%) 1 sided P = 0.003	**0/57 (0.0%)**

δ's represent differences between mean levels in each group and the placebo group, adjusted for baseline GCS scores using ANCOVA. 95% CI's are non-parametric bias-corrected and accelerated bootstrap confidence intervals (calculated using 2,000 replications).

The statistical significance of differences between groups in 15-day mortality rates and numbers of patients with erythema at injection site classified as moderate, intense or very intense are obtained using Fisher's exact test.

# denominator does not include individuals who were excluded from analyses

* with deaths coded as GCS = 3 and DRS coded as 30

** includes data on one individual who withdrew consent except for HIREOS & DRS

### Effect on all cause mortality

Vital status at two weeks following injury was known for 220 (96%) randomised patients. There were 31 (19.0%) deaths in those allocated Anatibant (all doses combined), compared with 9 (15.8%) among those allocated placebo (RR = 1.20 (0.61 to 2.36), 1 sided *p *= 0.38).

### Effect on Glasgow Coma Score

The mean GCS in patients allocated Anatibant (all doses combined) was 12.48 compared with 9.73 among those allocated placebo (a lower GCS signifies worse outcome). Adjusting for baseline GCS score the difference in the means (δ) was -0.55 [95% bootstrap CI (-1.42 to 0.59)], a difference that was not statistically significant (*p *> 0.05).

### Effect on Disability Rating Scale

The mean DRS among patients allocated Anatibant (all doses combined) was 11.18 compared with 9.73 among those allocated placebo (a higher DRS signifies a worse outcome). Adjusting for baseline GCS score the (non-statistically significant, *p *> 0.05) difference in the means (δ) was 1.61 [95% bootstrap CI (-1.16 to 4.23)].

### Effect on HIREOS

The mean HIREOS among patients allocated Anatibant (all doses combined) was 3.94 compared with 3.54 among those allocated placebo (a higher HIREOS signifies a worse outcome). Adjusting for baseline GCS score the (non-statistically significant, *p *> 0.05) difference in the means (δ) was 0.42 [95% bootstrap CI (-0.08 to 0.86)].

### Skin reactions at injection sites

Of patients who received Anatibant (all doses combined), 11.7% developed erythema at an injection site that was classified as moderate, intense or very intense. There were no such reactions in the placebo group (1 sided P = 0.003).

### Un-blinding

There was no emergency un-blinding of randomised patients.

## Discussion

### Principal findings

Our intention was to randomise 400 patients to this phase II trial of Anatibant in patients with traumatic brain injury. Had we done so, we would have had more than 90% power to detect a doubling in the number of patients with one or more SAEs among patients on active treatment. In fact, we were only able to randomise 228 patients and as a result the trial is underpowered. The statistically non-significant increase in the proportion of patients with at least one SAE that we observed is consistent with a sizeable increase or a modest reduction. We also observed statistically non-significant increases in mortality and morbidity.

### Strengths and limitations of the study

We conducted an international multi-centre randomised controlled trial of an emergency treatment for traumatic brain injury. Despite the well documented challenges of recruiting patients in emergency situations, patient recruitment was rapid and had it not been prematurely terminated the trial is likely to have reached its planned sample size of 400 patients. Allocation was well concealed through the use of central computerised telephone randomisation. Although the use of matching placebo helped to ensure that outcome assessment was blind to treatment allocation, the subcutaneous administration of Anatibant was associated with an increase in local skin reactions at the injection site which could have un-blinded treatment allocation in some cases. Skin reactions are unlikely to have biased assessment of all cause mortality, but the extent to which they might have biased the assessment of more subjective outcomes is open to question. There was minimal loss to follow up.

Although the TSC temporarily stopped recruitment in response to patient safety concerns from the DSMB, when the data on all randomised patients were reviewed, it was recommended that the trial could continue. Stopping recruitment until the data on all randomised patients could be represented to the DSMB, ensured that patient safety was the prime consideration. It would be inappropriate to conclude that this temporary stop was unjustified, since even extreme differences in patient outcome can occur by chance alone.

The primary outcome measure was the proportion of patients with one or more SAEs. In the context of critical illness however, the usual International Conference on Harmonisation Good Clinical Practice (ICH GCP) definition of an SAE [14] is problematic [15], since SAEs are the norm rather than the exception. For this reason, the ICH definition of SAE was made more context specific and medical occurrences which are expected in the course of the natural history of TBI (for example variations in intracranial pressure), or which are known to occur during routine diagnostic or therapeutic procedures (for example pain after surgery), were excluded. Our use of this revised definition may have resulted in some under-reporting. However, our approach to the reporting of SAEs in the context of critical illness has been used before [16]. Due to the decision to withdraw funding and the subsequent premature termination of the trial, this study was underpowered. It is unknown to the authors whether the decision to withdraw funding was influenced by the unscheduled un-blinded analyses.

### The BRAIN trial in relation to other trials

**Figure 2:**
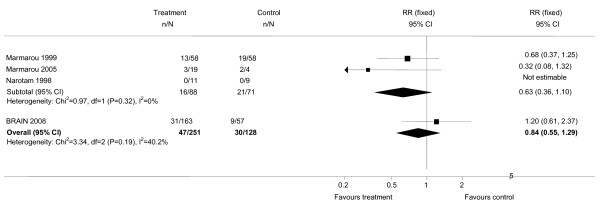
**Updated meta-analysis of effect of Bradykinin B2 receptor antagonists on mortality after traumatic brain injury**.

Although this is the largest trial of a Bradykinin B2 receptor antagonist in TBI conducted so far, it was small, and even in aggregate the total number of participants in clinical trials of B2 Receptor antagonists is less than 400 patients. The pooled relative risk for death from all trials combined is 0.84 (95% CI = 0.55 to 1.29) which is consistent with a modest increase or a modest decrease in the risk of death (Figure 2).

## Conclusion

Although with 228 patients randomised this trial was underpowered to detect even large increases in the risk of adverse events, it provides no reliable evidence of harm and no suspected unexpected serious adverse events (SUSARs) were reported.

The litigation delayed the conduct of the trial and incurred a considerable opportunity cost to the trial investigators. At a time when universities are encouraged to collaborate with industry in drug development the risk of litigation must be considered carefully.

## Abbreviations

BBB: blood-brain barrier; BB2: Bradykinin B2; BK: Bradykinin; CT scan: Computerised Tomography of the head; CI: confidence interval; DSMB: Data Safety Monitoring Board; DRS: Disability Rating Scale; GCS: Glasgow Coma Scale; HIREOS: Head Injury Related Early Outcome Score; ITT: intention to treat; IVRS: interactive voice response system; PK: Pharmacokinetic; PP: per protocol; RR: relative risks; SAE: serious adverse event; TBI: traumatic brain injury; TSC: Trial Steering Committee.

## Competing interests

This trial was developed and coordinated by LSHTM and was commercially funded by Xytis. The Independent TSC and DSMB were reimbursed costs incurred for meetings. The funder (Xytis) was involved through the Protocol Committee in the design of the trial. The collection of data was done by the collaborators listed and payments made were based on a per patient schedule. Monitoring of the trial data at site was done by monitors employed by the funder. The funder had view of the manuscript prior to submission and their comments were reviewed and accepted or rejected by the TSC.

## Disclaimer

The factual contents of this paper and the details provided about the clinical trial reported therein have not been verified by BioMed Central Limited ('the publisher') its agents or employees. This article has been published in accordance with this journal's obligation to report all clinical trials, in the public interest. No liability is accepted by the publisher, its agents or employees in connection with the contents of this paper.

## Authors' contributions

The trial was designed by HS and IR and the protocol was developed by the Protocol Committee and reviewed and accepted by the Trial Steering Committee. OF and CF were responsible for the data analysis. All authors were involved in the interpretation of the data and revised the manuscript critically for important intellectual content. All authors read and approved the submitted manuscript.
